# Disparities in maternal knowledge and practices on sleep training: a cross-sectional analysis of urban vs. rural areas

**DOI:** 10.3389/frsle.2025.1648131

**Published:** 2025-12-15

**Authors:** Kinandra Rafa Khalisha Rambey, Talitha Dinda Gunawan, Rini Sekartini

**Affiliations:** 1 Faculty of Medicine, Universitas Indonesia, Jakarta, Indonesia; 2 Department of Child Health, Faculty of Medicine, Universitas Indonesia, Jakarta, Indonesia

**Keywords:** maternal knowledge, maternal practice, sleep, sleep training, early childhood

## Abstract

**Background:**

Quality sleep is crucial for the growth and development of children. Sleep training is an effective method to improve the sleep quality of young children. This study aims to identify the knowledge and practices of mothers regarding sleep training for young children in Indonesia.

**Methods:**

This cross-sectional study included 417 mothers of children aged 3–36 months recruited purposively from three healthcare facilities in Indonesia, two urban and one rural, between March and June 2024. Data on sociodemographic characteristics, maternal knowledge, and maternal sleep-training behaviors were collected using validated, structured questionnaires. Item reliability and content validity were assessed through expert review and pilot testing. Descriptive, bivariate, and multiple linear regression analyses were performed using continuous knowledge and behavior scores, with co-sleeping variables included in sensitivity analyses. Mixed-effects linear models with random intercepts by site were applied to account for facility-level clustering and to assess model robustness.

**Results:**

Higher maternal education, formal employment, and higher socioeconomic status were significantly associated with greater knowledge and behavior scores. Mothers residing in urban areas demonstrated significantly better sleep-training practices, whereas knowledge scores did not differ between urban and rural participants. Multiple regression analyses confirmed that education level, employment type, and socioeconomic status independently predicted knowledge scores, while employment type, socioeconomic status, and urban residence independently predicted behavior scores. Mixed-effects modeling indicated that site-level clustering accounted for approximately 47% of variance in knowledge and 35% in behavior scores, with model comparisons (AIC/BIC) confirming robustness across specifications. Sensitivity analyses adjusting for co-sleeping and household crowding yielded consistent findings, indicating that the main associations were not affected by these contextual variables.

**Conclusion:**

Mothers with higher education, formal employment, and greater socioeconomic status demonstrated better knowledge and sleep-training behaviors; however, knowledge was not directly associated with practice. These findings highlight that improving maternal understanding alone may be insufficient to change behavior without addressing sociocultural norms—particularly widespread co-sleeping and household constraints—that influence sleep practices. Tailored, context-sensitive educational interventions are needed to promote consistent and developmentally appropriate sleep routines for young children in Indonesia.

## 1 Introduction

Sleep plays a vital role in the growth and development of children. Adequate and high-quality sleep is crucial for brain function, as well as for the child's mental and physical health. Fundamentally, sleep is a biological necessity that supports cellular regeneration, memory consolidation, and energy restoration. Children who receive sufficient sleep tend to exhibit better cognitive performance, emotional stability, and optimal physical development (Liu et al., 2022; Korownyk, 2018).

Sleep regulation in children develops rapidly, particularly during the first year of life, and continues to mature throughout childhood. In neonates, the absence of a circadian rhythm results in fragmented sleep patterns distributed across day and night, often interspersed with feeding. As circadian rhythms begin to establish, infants around 10–12 weeks of age typically begin to experience longer sleep periods at night. Between the ages of 1 and 4 years, children often continue to require daytime naps to meet their overall sleep needs (Galland et al., 2012; Meltzer et al., 2021; Liu, 2020; Li, 2023).

A child's sleep is shaped by a complex interplay of biological, environmental, behavioral, and social factors. Several studies have employed a socioecological framework to illustrate the various influences on children's sleep. These include individual-level (microsystem) factors such as genetics, age, ethnicity, medical and neurodevelopmental conditions, and behavior; family and school-level (mesosystem) factors such as parent-child relationships, parental mental health and stress levels, family routines, cultural beliefs, socioeconomic status, and school schedules; and broader environmental and sociocultural (macrosystem) influences including housing conditions, lighting, noise levels, community environments, and cultural norms (Galland et al., 2012; Honaker et al., 2021; Blunden et al., 2016; Hoyniak et al., 2022).

It is estimated that 20% to 40% of infants and school-aged children experience poor sleep quality, characterized by frequent nighttime awakenings, difficulty falling asleep, and reluctance to sleep independently. Sleep deprivation in children has been shown to negatively impact brain development and can lead to various health issues, including weakened immunity, obesity, and behavioral problems (Liu et al., 2022; Paruthi et al., 2016; Mindell and Williamson, 2018; Carter et al., 2014).

Sleep training is one approach that aims to promote more consistent and prolonged sleep in children, benefiting both the child and their caregivers. It encompasses a range of techniques and strategies designed to help children develop healthy sleep routines. Importantly, sleep training is not solely about teaching children to sleep independently—it also emphasizes age-appropriate sleep needs and routines. Factors such as a conducive sleep environment, daily routines, and pre-bedtime exposure to technology can significantly influence sleep patterns and the effectiveness of sleep training interventions (Korownyk, 2018; Liu, 2020; Li, 2023; Honaker et al., 2021).

Although sleep training is considered an effective method for improving children's sleep quality, little is known about the extent of maternal knowledge regarding this concept. This study seeks to explore mothers' understanding and awareness of sleep training and the extent to which these practices are implemented in daily life. Such insights are essential for identifying educational needs and informing health promotion strategies related to sleep in early childhood.

In the Indonesian context, research on sleep training remains limited, and the methods applied are often derived from Western cultural norms. Despite growing global attention to infant sleep practices, limited data exist on maternal understanding of sleep training in Indonesia. Existing studies also focused primarily on feeding and breastfeeding behavior, leaving a gap in parental knowledge regarding infant sleep patterns. Therefore, there is a significant gap in understanding how well-informed Indonesian mothers are about the importance of sleep and the role of sleep training. This study aims to assess mothers' knowledge and practice of sleep training and identify sociodemographic factors associated with the outcomes. Secondarily, this study also aims to examine the correlation between knowledge and practice of sleep training, which may guide future interventions and health education initiatives.

## 2 Materials and methods

This study utilized a cross-sectional design to assess maternal knowledge and practices regarding sleep training among mothers of children aged 3–36 months. A cross-sectional design was chosen to provide a descriptive overview of maternal knowledge and behavior in the current population and to explore associations among sociodemographic factors. Data collection was carried out between April and June 2024, or until the required sample size was reached. The study was conducted across three healthcare facilities in Indonesia: Jatinegara District Public Health Center and the Pediatric Outpatient Clinic of RSIA Bunda in East Jakarta (urban settings), and Keboan Public Health Center in Jombang Regency, East Java (a rural setting). The selection of facility was based on accessibility, patient volume, and willingness of the facilities to participate. These centers were chosen to capture variation in socioeconomic background and maternal exposure to health information. However, as these were facility-based samples, participating mothers may differ systematically from the general population. Mothers attending health centers are typically more health-conscious and have greater access to health services and health education. Therefore, the study sample may slightly overestimate levels of maternal knowledge and health-related practices compared to mothers who do not routinely visit health facilities.

The target population consisted of all mothers with children aged 3–36 months. The accessible population included mothers visiting the selected health facilities during the study period. Participants were selected using purposive sampling from among those who met the inclusion criteria. The sample size was calculated using a correlation-based (two-tailed) formula with a significance level (α) of 0.05, power (1–β) of 0.8, and an expected correlation coefficient of 0.144 (based on a similar study in China; Li, 2023). Calculation resulted in a required sample size of 376 participants. A total of 417 eligible respondents who met the inclusion criteria were then included in the final analysis to increase statistical power and account for potential missing data. This sample size exceeded the minimum requirement to ensure adequacy for correlation and multiple linear regression analyses.

Eligible participants were mothers with at least one child aged 3–36 months. If a mother had more than one child in this age range, the youngest was selected. Mothers of children with medical, developmental, or behavioral disorders—such as autism spectrum disorder (ASD), attention-deficit/hyperactivity disorder (ADHD), cerebral palsy, or Down syndrome—were excluded from the study.

Independent variables in this study included socioeconomic status (household income), residential area (urban or rural), maternal sociodemographic factors (age, education level, employment type), and the child's sleep location (own/shared bed or room). Socioeconomic status was classified based on monthly household income relative to the 2024 regional minimum wage. For urban participants (Jakarta), the threshold used was the *Upah Minimum Provinsi* (UMP) of Rp 5,067,381, while for rural participants (Jombang Regency), it was the *Upah Minimum Kabupaten* (UMK) of Rp 2,945,544. Participants with household incomes below these thresholds were categorized as “below minimum wage.” Education level was classified as “high” (senior high school or above) and “low” (junior high school or below). Maternal employment type was categorized as either formal or non-formal based on job characteristics. Formal employment included salaried positions with contracts and social security benefits, such as teachers, doctors, civil servants, industrial workers, private company employees, and state-owned enterprise employees. Non-formal employment encompassed jobs without formal contracts or social security, including freelance workers, street vendors, domestic helpers, and artists. The dependent variables were maternal knowledge of and behavior regarding sleep training practices.

Ethical approval for this study was obtained from the Research Ethics Committee of the Faculty of Medicine, Universitas Indonesia (KET-341/UN2.F1/ETIK/PPM.00.02/2024; protocol number of 24-02-0282). Informed consent was obtained from all participants following a clear explanation of the study's purpose and procedures. Participant anonymity and data confidentiality were strictly maintained.

Two structured questionnaires were used to assess maternal knowledge and sleep-training practices. These instruments were developed specifically for this study, as no prior validated questionnaires addressing sleep-training knowledge and behavior existed in the Indonesian context. Item generation was guided with previous cross-sectional study in China as reference (Li, 2023), and the items were then tailored to sleep training. The English and Indonesian versions of the instrument are provided in Supplementary Files S1, S2. The first questionnaire assessed maternal knowledge regarding sleep health and sleep training in children under 3 years of age. It consisted of 20 items, each scored on a five-point scale, resulting in a maximum possible score of 100. The second questionnaire evaluated maternal behavior related to the implementation of sleep training techniques. This instrument contained 12 items with multiple-answer options, each assigned specific point values based on relevance and effectiveness.

The questionnaire was reviewed by an expert in pediatric sleep, maternal health, and behavioral science to ensure content validity, increase clarity, and check for cultural appropriateness before data collection. A pilot study was conducted in a similar population with a total of 70 participants. A pilot study (*n* = 70) with mothers with similar sociodemographic characteristics to the main study population was conducted to evaluate the questionnaire. Item–total correlation analysis was performed to evaluate internal consistency reliability, identifying how strongly each item correlated with the overall scale. While strong correlations indicate coherence, this approach assesses reliability rather than construct validity. Cronbach's α values were 0.735 (knowledge) and 0.805 (practice), reflecting acceptable internal consistency. Some items with lower correlations were intentionally retained because they captured culturally specific behaviors (e.g., co-sleeping and night-feeding routines) considered essential to content validity (Supplementary Files S3, S4). The total scores were converted into percentages. Following conventions widely used in Indonesian public-health and KAP (knowledge–attitude–practice) research, a cutoff of 70% was applied to classify “good” knowledge or practice. This cut-off percentage has been adopted in several Indonesian studies, for instance, a KAP research in East Nusa Tenggara used ≥70% to define “good” knowledge, attitudes, and practices (Lee and Suryohusodo, 2022).

Data were analyzed using IBM SPSS version 25. Descriptive statistics were used to summarize respondent characteristics. Knowledge and behavior scores were analyzed as continuous scores. Bivariate analyses were performed using the independent samples *T*-test to see associations between sociodemographic factors and the scores, and the Spearman correlation test to see the association between the knowledge score and behavior score. Statistical significance was defined as *p* < 0.05. Multiple linear regression analysis was performed to estimate associations between sociodemographic factors and the scores, in addition, including co-sleeping factors (sharing a room with family member and sharing a bed with family member) to adjust for cultural context. Mixed-effects linear models with random intercepts by facility were compared with fixed-effects and cluster-robust models to test robustness to site clustering. Random slopes could not be estimated due to limited sample size. All analyses were based on complete-case data; no imputation was performed.

## 3 Results

### 3.1 Sociodemographic characteristics

A total of 417 mothers with children aged 3–36 months participated in this study. Data collection was conducted across three locations representing both urban and rural settings: Puskesmas Kecamatan Jatinegara, classified as a semi-urban area (53%); Puskesmas Keboan in Jombang Regency, a rural area (38.13%); and RSIA Bunda Menteng, located in an urban setting (8.87%). The data collection period spanned from March 27 to June 7, 2024. Participants were recruited directly on-site and completed a set of documents that included an identity form, an informed consent form, and two structured questionnaires. The majority of mothers were over 30 years old (50.6%), lived in urban areas (61.87%), had completed higher education (70.26%), were employed in the informal sector (88.25%), and had household incomes below the regional minimum wage (78.66%; Table 1).

**Table 1:** Distribution of maternal sociodemographic characteristics (*n* = 417).

**Characteristics**	**Frequency (%)**
**Age**	
≤ 30 years old	206 (49.4)
>30 years old	211 (50.6)
**Residential area**	
Urban	258 (61.87)
Rural	159 (38.13)
**Education level**	
High	293 (70.26)
Low	124 (29.74)
**Employment type**	
Formal	49 (11.75)
Informal	368 (88.25)
**Socioeconomic status**	
Minimum wage and above	89 (21.34)
Below minimum wage	328 (78.66)

### 3.2 Child characteristics and sleep environment

The majority of children in this study were male (50.84%), with a mean age of 19.28 ± 9.88 months, a median age of 19 months, and a range of 3–36 months. Regarding the sleep environment, most children (98.32%) shared both a sleeping space and a bed with others, and a significant proportion (82.49%) slept in their parents' bedroom. On average, 3.37 ± 1.02 individuals slept in the bedroom, with a median of 3 individuals, ranging from one person (the child alone) to eight individuals. Similarly, the average number of people sharing a bed with the child was 3.11 ± 0.98, with a median of 3 individuals, ranging from one person to seven individuals (Table 2).

**Table 2:** Child characteristics and sleep environment.

**Variables**	**Frequency (%) or Mean ±SD**
**Gender**	
Male	212 (50.84)
Female	205 (49.16)
Age (months)	19.28 ± 9.88
**Child's room and bed**	
Shared room and bed	410 (98.32)
Shared room, own bed	6 (1.44)
Own room and own bed	1 (0.24)
**Child's sleeping room**	
Parents' bedroom	344 (82.49)
Child's bedroom	30 (7.19)
Family room	42 (10.08)
Grandparents' bedroom	1 (0.24)

### 3.3 Maternal knowledge, sleep training behaviors, and sleep practices

Mothers demonstrated a mean knowledge score of 37.12 ± 16.5, with a median of 35 and a range of 0 to 85 out of a maximum score of 100. Meanwhile, the mean behavior score was 7.43 ± 2.02, with a median of 7 and a range of 3 to 15, out of a maximum score of 20.

In terms of children's sleep behaviors (Table 3), more than half of the children went to sleep (55.4%) and woke up (60.4%) at varying times each day. The majority (81.5%) had pre-sleep routines that included eating, such as drinking milk (41.7%), consuming healthy snacks (22.8%), or eating rice with side dishes (16.3%). Most parents (93.4%) reported performing hygiene-related routines before bedtime, including hand and foot washing (49.6%), tooth brushing (24.2%), and bathing (10.8%).

**Table 3:** Sleep-related behaviors among children aged 3–36 months.

**Sleep behavior**	**Frequency (%)**
**Bedtime routine**	
Consistent bedtime	186 (44.6)
Variable bedtime	231 (55.4)
**Wake-up routine**	
Consistent wake-up time	165 (39.6)
Variable wake-up time	252 (60.4)
**Pre-sleep eating**	
Healthy snacks	112 (22.8)
Milk	205 (41.7)
Rice-based meal	80 (16.3)
Sugary foods	4 (0.8)
No food intake	91 (18.5)
**Pre-sleep hygiene**	
Bathing	61 (10.8)
Brushing teeth	137 (24.2)
Washing hands and feet	280 (49.6)
Diaper change	47 (8.3)
Wiping body	3 (0.5)
No hygiene routine	35 (6.2)
Unknown	2 (0.4)
**Pre-sleep activities**	
Reading stories	72 (15.7)
Listening to soft music	93 (20.2)
Watching television	90 (19.6)
Using electronic devices	55 (12)
Physical exercise	7 (1.5)
Breastfeeding	76 (16.5)
Storytelling	5 (1.1)
Playing	47 (10.2)
Reciting prayers	1 (0.2)
No activity before sleep	12 (2.6)
Unknown	2 (0.4)
**Routines used to help the child fall asleep**	
Reading stories	30 (6.3)
Singing songs	50 (10.5)
Rocking	56 (11.8)
Breastfeeding	219 (46)
Playing	11 (2.3)
Watching television	2 (0.4)
Patting	13 (2.7)
Thumb sucking	1 (0.2)
No routine needed	92 (19.3)
Unknown	2 (0.4)
**Presence of others in the child's sleeping room**	
Sleeping in the same room with family members	413 (99%)
Sleeping in the same room with caregivers	2 (0.5)
Sleeping alone in the room	2 (0.5)
**Presence of others sharing the same bed**	
Sharing a bed with family members	408 (97.8)
Sharing a bed with caregivers	8 (1.9)
Sleeping alone in the bed	1 (0.2)
**Child's bedroom lighting**	
Light on	184 (44.12)
Light off	231 (55.4)
Unknown	2 (0.48)
**Child's bedroom cooling**	
Air conditioner (AC)	83 (19.9)
Electric fan	318 (76.26)
No cooling device	16 (3.84)
**Entertainment in the child's bedroom**	
Favorite doll or toy	241 (55.3)
Television	74 (17)
Electronic devices	7 (1.6)
Book	2 (0.5)
No entertainment in the bedroom	110 (25.2)
Unknown	2 (0.5)
**Parental response when the child wakes up crying**	
Rocking the child	99 (21.6)
Singing	27 (5.9)
Breastfeeding	266 (58.1)
Patting the child	46 (10)
Asking the child what's wrong	3 (0.7)
Playing with the child	1 (0.2)
Watching television	1 (0.2)
Reciting prayers	1 (0.2)
Giving the child water	2 (0.4)
No response	8 (1.7)
Unknown	4 (0.9)
**Methods used to put the child to sleep**	
Breastfeeding	311 (62.1)
Rocking	82 (16.4)
Reading stories	24 (4.8)
Alternating sleep methods	50 (10)
No specific method	34 (6.8)

A large proportion of children (97%) engaged in pre-sleep activities, primarily listening to soothing music (20.2%), watching television (19.6%), or breastfeeding (16.5%). Most children (80.3%) had specific sleep onset routines, such as breastfeeding (46%), being rocked (11.8%), or being sung to (10.5%).

Nearly all children (99%) shared a bedroom with family members, and most (97.8%) also shared a bed. Regarding bedroom characteristics, most children slept with the lights off (55.4%), used a fan for cooling (76.26%), and had sleep-related items such as stuffed animals or favorite toys (55.3%), a television (17%), or a gadget (1.6%) in the room.

A majority of parents (97.4%) responded to their child's nighttime awakenings with soothing actions, primarily breastfeeding (58.1%), rocking (21.6%), patting (10%), or singing (5.9%). Additionally, 93.2% of parents reported using various methods to help their child fall asleep, including breastfeeding (62.1%), rocking (16.4%), and storytelling (4.8%). About 10% of parents alternated between different methods to help their child sleep (Table 3).

### 3.4 Association between sociodemographic characteristics and maternal knowledge of sleep training

Independent sample *T* test showed that high education level, formal employment, and better socioeconomic status were significantly associated with knowledge scores, and no significant association between age groups and residential areas (Table 4).

**Table 4:** Association between sociodemographic characteristics and maternal knowledge of sleep training score (*n* = 417).

**Characteristics**	**Knowledge score Mean ±SD**	***t* (df)**	***p* value**	**Mean difference (95% CI)**
**Maternal age**		0.97 (417)	0.335	1.56 (−1.61, −4.73)
≤ 30 years old	38.00 ± 16.44			
>30 years old	36.45 ± 16.60			
**Residential area**		−0.55 (402.92)	0.580	−0.86 (−3.91, 2.19)
Rural	36.69 ± 13.49			
Urban	37.55 ± 18.15			
**Education level**		−7.21 (259)	< 0.001^*^	−11.54 (−14.69, −8.39)
Low	29.12 ± 14.45			
High	40.66 ± 16.15			
**Employment type**		−4.02 (59.55)	< 0.001^*^	−10.87 (−16.28, −5.47)
Informal	35.92 ± 15.87			
Formal	46.80 ± 18.18			
**Socioeconomic status**		−4.57 (135.89)	< 0.001^*^	−9.65 (−13.83, −5.48)
Below minimum wage	34.94 ± 14.87			
Minimum wage and above	44.60 ± 19.30			

^*^Statistically significant at p < 0.05.

### 3.5 Association between sociodemographic characteristics and maternal sleep training practices

Independent sample *T* test showed that urban residence, high education level, formal employment, and better socioeconomic status were significantly associated with behavior scores, and no significant association between age groups (Table 5).

**Table 5:** Association between sociodemographic characteristics and maternal sleep training behavior score (*n* = 417).

**Characteristics**	**Behavior score Mean ±SD**	***t* (df)**	***p* value**	**Mean difference (95% CI)**
**Maternal age**		0.879 (417)	0.380	0.173 (−0.214, 0.560)
≤ 30 years old	7.51 ± 1.90			
>30 years old	7.34 ± 2.12			
**Residential area**		−3.904 (357.10)	< 0.001^*^	−0.764 (−1.149, −0.379)
Rural	6.95 ± 1.88			
Urban	7.71 ± 2.04			
**Education level**		−2.697 (253.92)	0.007^*^	−0.556 (−0.963, −0.150)
Low	7.03 ± 1.879			
High	7.59 ± 2.052			
**Employment type**		−4.12 (61.21)	< 0.001^*^	−1.292 (−1.91, −0.66)
Informal	7.27 ± 1.95			
Formal	8.56 ± 2.09			
**Socioeconomic status**		−4.07 (152.46)	< 0.001^*^	−0.968 (−1.43, −0.498)
Below minimum wage	7.19 ± 1.933			
Minimum wage and above	8.16 ± 2.108			

^*^Statistically significant at p < 0.05.

### 3.6 Association between maternal knowledge and sleep training practices

Spearman correlation test between the continuous scores of both knowledge and behavior revealed a weak relationship, with ρ of 0.275 and *p* < 0.001.

### 3.7 Multiple linear regression analysis of factors associated with maternal knowledge and sleep training practices

Continuous scores of both knowledge and behavior were analyzed using multiple linear regression. The results showed that education level, employment type, and socioeconomic status were independently associated with knowledge scores (*p* < 0.001) after adjustment for maternal age and residential area. Participants with high education level scored 9.29 points higher on their knowledge scores compared to those with low education level. Formally employed participants scored 6.26 points higher compared to those informally employed, while participants with income at or above UMR scored 6.06 points higher compared to those with income below UMR on their knowledge scores (Table 6). The model explained approximately 14.9% (adjusted *R*^2^ = 0.138) of the variance in knowledge score, and no multicollinearity was observed [all variance inflation factor (VIF) < 1.2].

**Table 6:** Multiple linear regression analysis on factors associated with maternal knowledge of sleep training.

**Variable**	**β (Unstandardized)**	**SE**	**β (Standardized)**	***t***	***p*-value**	**VIF**
(Constant)	28.865	1.810	–	15.94	< 0.001^*^	–
Maternal age (>30 years old vs. ≤ 30 years old)	−0.565	1.508	−0.017	−0.38	0.708	1.013
Residential area (Urban vs. rural)	−0.116	1.551	−0.003	−0.08	0.940	1.012
Education level (High vs. low)	9.292	1.710	0.258	5.44	< 0.001^*^	1.091
Employment type (Formal vs. informal)	6.361	2.411	0.125	2.64	0.009^*^	1.089
Socioeconomic status (At or above UMR vs. below UMR)	6.059	1.860	0.156	3.26	0.001^*^	1.113

^*^Model summary: R = 0.386, R^2^ = 0.149, Adjusted R^2^ = 0.138, F_(5, 413)_ = 14.43, and p < 0.001 with no multicollinearity detected (all VIF < 1.2).

Residential area, employment type, and socioeconomic status of participants were independently associated with behavior scores after adjustment for maternal age and education level. Mothers residing in urban areas had 0.71 points higher on behavior scores compared to those in rural areas, and those with income at or above UMR had 0.67 points higher on behavior scores compared to those with income below UMR. Formally employed participants had 0.99 points higher on behavior scores compared to those employed informally (Table 7). The model explained approximately 9.0% (adjusted *R*^2^ = 0.090) of the variance in practice scores, and all collinearity diagnostics were within acceptable limits (VIF < 1.2).

**Table 7:** Multiple linear regression analysis on factors associated with maternal sleep training behavior.

**Variable**	**β (Unstandardized)**	**SE**	**β (Standardized)**	**t**	***p*-value**	**VIF**
(Constant)	6.636	0.227	–	29.23	< 0.001^*^	–
Maternal age (>30 years old vs. ≤ 30 years old)	−0.152	0.189	−0.038	−0.81	0.421	1.013
Residential area (Urban vs. rural)	0.711	0.194	0.172	3.66	< 0.001^*^	1.012
Education level (High vs. low)	0.210	0.214	0.048	0.98	0.328	1.091
Employment type (Formal vs. informal)	0.987	0.302	0.159	3.27	0.001^*^	1.089
Socioeconomic status (At or above UMR vs. below UMR)	0.670	0.233	0.141	2.87	0.004^*^	1.113

^*^Model summary: R = 0.318, R^2^ = 0.101, Adjusted R^2^ = 0.090, F_(5, 413)_ = 9.304, and p < 0.001, with no multicollinearity detected (all VIF < 1.2).

Sensitivity analyses were conducted to examine whether cultural context of co-sleeping, as measured by sharing a room with family member and sharing a bed with family member, might influence the associations between sociodemographic factors and knowledge and behavior scores. The results indicated that including these cultural context variables did not change the significant associations between the sociodemographic factors with knowledge score and behavior score, and the co-sleeping factors themselves were not statistically significant (Tables 8, 9).

**Table 8:** Sensitivity analysis on co-sleeping variables as covariate in association with maternal knowledge of sleep training.

**Variable**	**β (Unstandardized)**	**SE**	**β (Standardized)**	**t**	***p*-value**	**VIF**
(Constant)	60.256	11.173	–	5.393	< 0.001^*^	–
Maternal age (>30 years old vs. ≤ 30 years old)	−1.061	1.501	−0.032	−0.707	0.480	1.025
Residential area (Urban vs. rural)	−0.493	1.541	−0.015	−0.320	0.749	1.020
Education level (High vs. low)	9.063	1.694	0.251	5.350	< 0.001^*^	1.093
Employment type (Formal vs. informal)	6.907	2.392	0.136	2.887	0.004^*^	1.094
Socioeconomic status (At or above UMR vs. below UMR)	4.735	1.898	0.122	2.495	0.013^*^	1.183
Room sharing with family member (No vs. Yes)	−19.661	12.400	−0.082	−1.586	0.114	1.329
Bed sharing with family member (No vs. Yes)	−11.145	6.451	−0.092	−1.728	0.085	1.418

^*^Model summary: R = 0.412, R^2^ = 0.170, Adjusted R^2^ = 0.156, F_(7, 411)_ = 12.022, and p < 0.001, with no multicollinearity detected (all VIF < 1.5).

**Table 9:** Sensitivity analysis on co-sleeping variables as covariate in association with maternal sleep training behavior.

**Variable**	**β (Unstandardized)**	**SE**	**β (Standardized)**	**t**	***p*-value**	**VIF**
(Constant)	9.760	1.409	–	6.925	< 0.001^*^	–
Maternal age (>30 years old vs. ≤ 30 years old)	−0.194	0.189	−0.048	−1.025	0.306	1.025
Residential area (Urban vs. rural)	0.681	0.194	0.164	3.501	0.001^*^	1.020
Education level (High vs. low)	0.191	0.214	0.043	0.894	0.372	1.093
Employment type (Formal vs. informal)	1.036	0.302	0.167	3.433	0.001^*^	1.094
Socioeconomic status (At or above UMR vs. below UMR)	0.562	0.239	0.119	2.349	0.019^*^	1.183
Room sharing with family member (No vs. Yes)	−2.372	1.564	−0.081	−1.516	0.130	1.329
Bed sharing with family member (No vs. Yes)	−0.704	0.814	−0.048	−0.866	0.387	1.418

^*^Model summary: R = 0.337, R^2^ = 0.113, Adjusted R^2^ = 0.098, F_(7, 411)_ = 7.498, and p < 0.001, with no multicollinearity detected (all VIF < 1.5).

### 3.8 Mixed model analysis accounting different recruitment sites

A linear mixed-effect model was performed to assess whether site-level clustering affected knowledge and behavior scores. The mean knowledge score was 43.29 (95% CI: 7.76–78.83, *p* = 0.035). The estimated variance between sites was 195.51, and the within-site residual variance was 222.90, resulting in an intraclass correlation coefficient (ICC) of 0.47 (Table 10). The mean behavior score was 7.96 (95% CI: 4.40–11.52, *p* = 0.011). The estimated variance between sites was 1.91, and the residual variance between sites was 3.58, resulting in an ICC of 0.35 (Table 11). The direction and statistical significance of key predictors remained consistent, and model fit values (AIC/BIC) were comparable across specifications (ΔAIC < 3), suggesting that the findings are robust to clustering.

**Table 10:** Mixed model analysis to account for site-clustering on maternal knowledge of sleep training.^*^

**Component**	**Estimate**	**Std. Error**	***p value***	**95% CI**
Fixed effect (intercept)	43.29	8.13	0.035	7.76–78.83
Random effect (intercept variance between sites)	195.51	199.88	0.328	26.36–1450.11
Residual (within-site variance)	222.90	15.46	< 0.001	194.58–255.35

^*^Model fit: −2 Log Likelihood = 3461.2, AIC = 3465.2, and BIC = 3473.271.

**Table 11:** Mixed model analysis to account for site-clustering on maternal sleep training behavior.^*^

**Component**	**Estimate**	**Std. Error**	***p value***	**95% CI**
Fixed effect (intercept)	7.961	0.806	0.011	4.40–11.52
Random effect (intercept variance betweensites)	1.906	1.976	0.335	0.25–14.55
Residual (within-site variance)	3.577	0.248	< 0.001	3.12–4.10

^*^Model fit: −2 Log Likelihood = 1732.93, AIC = 1736.93, and BIC = 1745.001.

## 4 Discussion

This study revealed that mothers' knowledge regarding sleep training for children aged 3 to 36 months was generally suboptimal. This aligns with previous research showing that many parents lack an accurate understanding of children's sleep requirements, particularly in communities with limited access to pediatric health information (Mindell and Williamson, 2018; Wilson et al., 2014). Mothers with higher education levels, formal employment, and monthly incomes above the regional minimum wage (UMR) demonstrated significantly higher knowledge scores. Similarly, those residing in urban areas, with higher education, formal employment, and higher income, exhibited significantly better behavioral scores. These findings may reflect the greater access to health information, medical services, and educational resources among individuals of higher socioeconomic status. Nevertheless, the implementation of sleep training is likely influenced not only by sociodemographic factors but also by contextual elements such as limited living space, household norms, and cultural values shared across both urban and rural settings (Ordway et al., 2020; Abuhammad et al., 2024).

Overall, the study did not identify a significant association between maternal knowledge and sleep training behavior. This indicates that higher knowledge does not necessarily lead to better practice. This result is consistent with previous findings suggesting that knowledge alone is often insufficient to drive behavioral change. According to Bronfenbrenner's ecological systems theory, maternal behavior is not shaped by individual knowledge in isolation but is formed through complex interactions involving individual, family, social, cultural, and broader societal factors. Variables such as home environment, parenting norms related to sleep (for example, co-sleeping), caregiver stress, and psychosocial conditions can affect children's sleep patterns and mothers' ability to apply structured sleep routines (Lee and Suryohusodo, 2022; Ordway et al., 2020) Multiple linear regression analysis demonstrated that formal employment type and better socioeconomic status were independently associated with both knowledge and behavior scores. Higher education level was independently associated with knowledge scores, while urban residence was independently associated with behavior scores, indicating that those sociodemographic factors may motivate either knowledge or behavior among mothers. Besides that, these findings also need to be acknowledged with cultural and structural constraints in mind (Mindell and Williamson, 2018; Mindell et al., 2010).

A prominent cultural pattern observed in this study was the high prevalence of co-sleeping. A total of 99 percent of children were reported to sleep in the same room, and 97.8 percent shared the same bed with a family member. This practice reflects normative Indonesian and Southeast Asian values emphasizing emotional closeness and family interdependence, which fundamentally conflict with the independent sleep goals promoted by typical Western sleep training strategies (Pal et al., 2023; Mindell et al., 2010; Mason et al., 2021). However, this practice is also linked to the formation of negative sleep associations, in which children become reliant on specific stimuli such as breastfeeding, rocking, or singing to fall asleep or return to sleep after night awakenings, which leads to the high rates of irregular sleep and wake schedules. The deeply embedded family norms and potentially limited living space, where solitary infant sleep is not culturally valued or structurally feasible, may explain this. These associations may also interfere with the development of independent sleep regulation or self-soothing abilities. To reduce such dependencies, it is recommended that children be placed in bed while still awake after feeding (Sambo et al., 2010; Garrido et al., 2024; Abdul Jafar et al., 2021). Despite that, in this study, sensitivity analyses showed that the primary associations between sociodemographic factors and the scores are robust, and not affected by cultural context of room-sharing and bed-sharing with family member. This might be explained by the minimal variation in co-sleeping practices across households, limiting its impact on the scores.

Additional factors associated with poor sleep patterns included irregular sleep and wake schedules and exposure to digital devices or television before bedtime (Fadzil, 2021; Mindell et al., 2021). More than half of the children in this study lacked regular bedtime routines. Such irregularities can disrupt the child's circadian rhythm and negatively affect overall sleep quality. Furthermore, screen exposure before bedtime has been shown to delay sleep onset and reduce sleep duration due to cognitive stimulation and blue light, which suppresses melatonin secretion (Staples et al., 2021; Pickard et al., 2024). In contrast, calm and consistent bedtime routines such as reading, singing, or taking a warm bath before sleep at the same time each night have been shown to significantly improve sleep quality in young children (Mindell and Williamson, 2018).

Based on the mixed-model analyses accounting for site clustering, the ICC was relatively high, indicating some clustering of maternal knowledge and behaviors within sites, possibly reflecting shared exposure to local health promotion efforts or cultural norms. However, sensitivity analyses using mixed-effects models with random intercepts produced consistent results. The overall findings of this study indicate that inadequate sleep training practices may contribute to the high prevalence of sleep disturbances among infants and toddlers in primary healthcare facilities in Indonesia. Previous research conducted in Jakarta reported that approximately 66 percent of children aged 1 to 36 months experienced sleep problems, with an average of two nighttime awakenings per night. Poor sleep hygiene during infancy is associated with long-term consequences, including behavioral problems, emotional dysregulation, and developmental delays. It may also adversely impact parental wellbeing by increasing maternal stress, fatigue, and symptoms of depression (Sambo et al., 2010; Muller and Guse, 2024).

These findings underscore the importance of developing educational interventions that not only improve maternal knowledge of sleep training but also address cultural, social, and structural barriers to implementation. Culturally responsive, contextually appropriate, and evidence-based approaches are essential to promoting healthy sleep routines in young children. When effectively delivered, such interventions may serve as a long-term strategy to support child development and enhance the wellbeing of families.

## 5 Limitations

This study has several limitations that should be considered when interpreting the findings. First, although the maternal knowledge and behavior questionnaires were developed and tested for both validity and reliability, they were based on self-reported data. This introduces the potential for social desirability bias, wherein participants may respond with answers they perceive as more acceptable or ideal, rather than reflecting their actual knowledge or behaviors.

Second, while the questionnaires consisted of structured, multiple-choice items, open-ended options (“other: ____”) were also included to allow participants to express individualized responses. In cases where responses aligned with evidence-based practices, they were scored accordingly. However, because not all participants utilized these open-ended options—and these responses were not part of the standardized choices—this may have affected consistency in scoring and the comparability of responses. Moreover, certain valid answers provided in the open fields may not have been captured or reflected in the overall scoring, potentially leading to underrepresentation of participant knowledge or behavior that fell outside predefined categories. The instruments demonstrated acceptable reliability and face/content validity through expert review and pilot testing. Item–total correlations supported internal coherence, but further construct validation, such as factor analysis and external convergence tests, should be pursued in future studies to strengthen psychometric evidence and cross-cultural applicability.

Third, the cross-sectional design of the study limits the ability to draw causal conclusions. While the data allow for the identification of associations between maternal knowledge and sleep training behavior, no inferences about directionality or long-term outcomes can be made. Longitudinal research is needed to explore how maternal knowledge and practices evolve and affect sleep outcomes in children.

Although efforts were made to include participants from both urban and rural areas, the findings may not be fully generalizable to all populations in Indonesia. The use of purposive sampling from selected health facilities may have introduced selection bias, and participants may differ systematically from mothers in other regions or settings, particularly those with different cultural, economic, or healthcare access profiles. Mixed model analysis to account for site-level clustering showed that approximately 47% of the total variance in knowledge scores and approximately 35% of the variance in behavior scores was attributable to differences between sites.

Lastly, this study focused specifically on maternal knowledge and behavior, and did not examine other potentially influential factors such as paternal involvement, family dynamics, cultural norms, or maternal mental health. Future studies should consider a more comprehensive approach to capture the complex interplay of factors influencing sleep training practices in early childhood.

## 6 Conclusion

This study highlights significant disparities in maternal knowledge and practices related to sleep training between urban and rural areas in Indonesia. Sleep training knowledge and behaviors across both groups remained suboptimal and not correlated. This suggests that knowledge alone may be insufficient to drive behavior change, especially when cultural norms such as co-sleeping, overcrowded sleep environments, and limited access to reliable health information are present.

The widespread prevalence of poor maternal knowledge and inadequate sleep training practices underscores the need for targeted health education interventions. These efforts should move beyond information dissemination and be tailored to the sociocultural realities of Indonesian families. Emphasizing structured bedtime routines, minimizing dependence on sleep associations, and promoting consistent sleep schedules are key strategies. Future programs should adopt culturally sensitive and ecologically informed approaches to more effectively support healthy sleep development in young children.

## Data Availability

The raw data supporting the conclusions of this article will be made available by the authors, without undue reservation.

## References

[B1] Abdul Jafar N. K. Tham E. K. H. Pang W. W. Fok D. Chua M. C. Teoh O. H. . (2021). Association between breastfeeding and sleep patterns in infants and preschool children. Am. J. Clin. Nutr. 114, 1986–1996. doi: 10.1093/ajcn/nqab297 34582549

[B2] Abuhammad S. Younis A. B. Ahmed A. H. (2024). Impact of a structured sleep education program on mothers' knowledge and attitudes toward infant sleeping. Heliyon 10:e29885. doi: 10.1016/j.heliyon.2024.e29885 38711628 PMC11070819

[B3] Blunden S. Etherton H. Hauck Y. (2016). Resistance to cry intensive sleep intervention in young children: are we ignoring children's cries or parental concerns? Children 3:8. doi: 10.3390/children3020008 27417246 PMC4934563

[B4] Carter K. A. Hathaway N. E. Lettieri C. F. (2014). Common sleep disorders in children. Am. Fam. Phys. 89, 368–377. 24695508

[B5] Fadzil A. (2021). Factors affecting the quality of sleep in children. Children 8:122. doi: 10.3390/children8020122 33572155 PMC7915148

[B6] Galland B. C. Taylor B. J. Elder D. E. Herbison P. (2012). Normal sleep patterns in infants and children: a systematic review of observational studies. Sleep Med. Rev. 16, 213–222. doi: 10.1016/j.smrv.2011.06.001 21784676

[B7] Garrido F. González-Caballero J. L. García P. Gianni M. L. Garrido S. González L. . (2024). Association between co-sleeping in the first year of life and preschoolers' sleep patterns. Eur. J. Pediatr. 183, 2111–2119. doi: 10.1007/s00431-024-05429-2 38351212 PMC11035441

[B8] Honaker S. M. Mindell J. A. Slaven J. E. Schwichtenberg A. J. (2021). Implementation of infant behavioral sleep intervention in a diverse sample of mothers. Behav. Sleep Med. 19, 547–561. doi: 10.1080/15402002.2020.1817745 32954835 PMC13112062

[B9] Hoyniak C. P. Bates J. E. Camacho M. C. McQuillan M. E. Whalen D. J. Staples A. D. . (2022). The physical home environment and sleep: what matters most for sleep in early childhood. J. Fam. Psychol. 36, 757–769. doi: 10.1037/fam0000977 35266772 PMC9747092

[B10] Korownyk C. (2018). Sleep training: rest easy? *Can. Fam. Phys*. 64:41. PMC5962992 29358251

[B11] Lee F. Suryohusodo A. A. (2022). Knowledge, attitude, and practice assessment toward COVID-19 among communities in East Nusa Tenggara, Indonesia: a cross-sectional study. Front. Public Health 10:957630. doi: 10.3389/fpubh.2022.957630 36388283 PMC9659730

[B12] Li D. (2023). Characterization of parental knowledge on early child sleep and association with child sleep quality: a cross-sectional pilot study in Chongqing, China. Risk Manag. Healthc. Policy 16, 851–864. doi: 10.2147/RMHP.S408428 37197563 PMC10183354

[B13] Liu A. (2020). Sleep training. Pediatr. Ann. 49, e101–e105. doi: 10.3928/19382359-20200218-01 32155274

[B14] Liu J. Ji X. Pitt S. Wang G. Rovit E. Lipman T. . (2022). Childhood sleep: physical, cognitive, and behavioral consequences and implications. World J. Pediatr. 23, 1–11. doi: 10.1007/s12519-022-00647-w 36418660 PMC9685105

[B15] Mason G. M. Holmes J. F. Andre C. Spencer R. M. C. (2021). Bedsharing in early childhood: frequency, partner characteristics, and relations to sleep. J. Genet. Psychol. 182, 269–288. doi: 10.1080/00221325.2021.1916732 33988085 PMC8522355

[B16] Meltzer L. J. Williamson A. A. Mindell J. A. (2021). Pediatric sleep health: it matters, and so does how we define it. Sleep Med. Rev. 57:101425. doi: 10.1016/j.smrv.2021.101425 33601324 PMC9067252

[B17] Mindell J. A. Kuhn B. Lewin D. S. Meltzer L. J. Sadeh A. American Academy of Sleep Medicine (2021). Behavioral treatment of bedtime problems and night wakings in infants and young children. Children 8:122. 17068979

[B18] Mindell J. A. Sadeh A. V. Wiegand B. How T. H. Goh D. Y. T. (2010). Cross-cultural differences in infant and toddler sleep. Sleep Med. 11, 274–280. doi: 10.1016/j.sleep.2009.04.012 20138578

[B19] Mindell J. A. Williamson A. A. (2018). Benefits of a bedtime routine in young children: sleep, development, and beyond. Sleep Med. Rev. 40, 93–108. doi: 10.1016/j.smrv.2017.10.007 29195725 PMC6587181

[B20] Muller J. Guse T. (2024). The influence of infant sleep problems and sleep training on maternal subjective well-being. Health SA 29:2660. doi: 10.4102/hsag.v29i0.2660 39114343 PMC11304104

[B21] Ordway M. R. Sadler L. S. Jeon S. O'Connell M. Banasiak N. Fenick A. M. . (2020). Sleep health in young children living with socioeconomic adversity. Res. Nurs. Health 43, 329–340. doi: 10.1002/nur.22023 32306413 PMC7368803

[B22] Pal E. Blackwell J. E. Ball H. L. Collings P. J. (2023). Sociodemographic, temporal and bedtime routine correlates of sleep timing and duration in South Asian and white children: a Born in Bradford study. Sleep Med. X 5:100068. doi: 10.1016/j.sleepx.2023.100068 37033692 PMC10074244

[B23] Paruthi S. Brooks L. J. D'Ambrosio C. Hall W. A. Kotagal S. Lloyd R. M. . (2016). Recommended amount of sleep for pediatric populations: a consensus statement of the American Academy of Sleep Medicine. J. Clin. Sleep Med. 12, 785–786. doi: 10.5664/jcsm.5866 27250809 PMC4877308

[B24] Pickard H. Chu P. Essex C. Goddard E. J. Baulcombe K. Carter B. . (2024). Toddler screen use before bed and its effect on sleep and attention. JAMA Pediatr. 178, 1270–1279. doi: 10.1001/jamapediatrics.2024.3997 39432278 PMC11581737

[B25] Sambo C. M. Sekartini R. Trihono P. P. (2010). Sleep patterns in 1 to 36 month-old children. Paediatr. Indones. 50, 170–175. doi: 10.14238/pi50.3.2010.170-5

[B26] Staples A. D. Hoyniak C. McQuillan M. E. Molfese V. Bates J. E. (2021). Screen use before bedtime: consequences for nighttime sleep in young children. Infant. Behav. Dev. 62:101522. doi: 10.1016/j.infbeh.2020.101522 33385752 PMC7977486

[B27] Wilson K. E. Miller A. L. Bonuck K. Lumeng J. C. Chervin R. D. (2014). Evaluation of a sleep education program for low-income preschool children and their families. Sleep 37, 1117–1125. doi: 10.5665/sleep.3774 24882907 PMC4015386

