# Supplementary material for: Disparities in maternal knowledge and practices on sleep training: a cross-sectional analysis of urban vs. rural areas
Source: Front Sleep. 2025 Dec 15;4:1648131. doi: 10.3389/frsle.2025.1648131 (PMC12713900; doi:10.3389/frsle.2025.1648131)
Supplement: Supplementary file 1 [file Data_Sheet_1.docx]

**Supplementary File 1.** Questionnaire Titled “Knowledge and Behavior of Mothers with Children Aged 3–36 Months Regarding Sleep Training”

**RESEARCH SUBJECT IDENTITY FORM**

**Date of Completion:**

**Location of Completion*:** Jatinegara Community Health Center / Bunda Maternity Hospital Clinic / Keboan Community Health Center

1. **Mother’s Identity**

| a. Name | : | | |
| --- | --- | --- | --- |
| b. Place, date of birth | : | | |
| c. Age | : | | |
| d. Address | : | | |
| e. Phone number | : | | |
| f. Highest education level** : | | 1. Elementary 2. Junior High 3. Senior High 4. Diploma | 1. Bachelor’s degree 2. Master’s degree 3. Doctoral degree 4. No formal education |

g. Occupation :

h. Pendapatan keluarga** : **For residents of DKI Jakarta***

- 1. Less than IDR 5,000,000/month
  2. IDR 5,000,000/month or moire

1. **Childs Identity**

**For residents of Jombang Regency***

1. Less than IDR 3,000,000/month
2. IDR 3,000,000/month or more
   1. Name :
   2. Sex :
   3. Place, date of birth :
   4. Age :
   5. Child number : out of siblings
3. **Notes**

- : Cross out if not applicable

**: Circle the appropriate choice

1. **Child’s Sleeping Room**

Circle and fill in the appropriate answer according to your child’s sleeping conditions at home. You may choose more than one answer.

1. My child’s sleeping room is:
   (a) Child’s bedroom
   (b) Parents’ bedroom
   (c) Living room
   (d) Others, specify: ___________________________
2. My child sleeps in the same room with:
   (a) Alone
   (b) Mother
   (c) Father
   (d) Sibling(s), number of siblings: _____
   (e) Others, specify: ____________, number of people: _____
   **Total number of people in one room = _____**
3. My child sleeps on the same bed with:
   (a) Alone
   (b) Mother
   (c) Father
   (d) Sibling(s), number of siblings: _____
   (e) Others, specify: ____________, number of people: _____
   **Total number of people on one bed = _____**

**KNOWLEDGE QUESTIONS**

Choose one correct answer for each question.

1. Sleep training is a strategy that can support independent sleep in children. Which statement is TRUE?
   a. Sleep training can solve sleep problems in children
   b. Sleep training can help establish a consistent bedtime routine
   c. Sleep training is not the same for every child—it should be adjusted to the child’s and parents’ preferences
   d. Parents’ anxiety when hearing their child cry can hinder success
   e. All of the above
2. What is the best age to start sleep training?
   a. 1–3 months
   b. 4–6 months
   c. 7–9 months
   d. 10–12 months
   e. 1–3 years
3. Which of the following is NOT a sign that a child is ready for sleep training?
   a. The child does not cry easily when away from parents
   b. The child begins to have regular sleep/wake cycles
   c. The child can self-soothe to sleep
   d. The child can sleep through the night without feeding
   e. The child has a mature internal sleep regulation system
4. The goal of sleep training is to help the child achieve independent sleep through positive behaviors. Which of the following supports this goal?
   a. Rocking the child to sleep
   b. Singing before bed
   c. Breastfeeding before bed
   d. The child lying beside parents
   e. The child sleeping with a favorite doll
5. Which statement is TRUE about children’s sleep?
   a. Allowing a child to cry to sleep can cause emotional problems later
   b. There is no evidence that sleep training improves parents’ physical and mental health
   c. Children should be breastfed until asleep to ensure deep sleep
   d. Parents should rock the child until asleep to prevent night wakings
   e. None of the above
6. The method where the parent puts the baby to bed and leaves the room without checking until morning is known as:
   a. Faded bedtime
   b. Cry-it-out
   c. Scheduled awakenings
   d. Controlled crying
   e. Camping-out
7. The method where parents leave the room and check the baby at increasing intervals (3, 5, 10 minutes) over one week is:
   a. Cry-it-out
   b. Faded bedtime
   c. Scheduled awakenings
   d. Controlled crying
   e. Camping-out
8. The method that delays bedtime and implements bedtime routines so the child falls asleep faster is:
   a. Camping-out
   b. Scheduled awakenings
   c. Faded bedtime
   d. Controlled crying
   e. Cry-it-out
9. The method where parents gently wake the child (e.g., tickling feet) 15–30 minutes before the usual waking time so they can resettle quickly is:
   a. Faded bedtime
   b. Cry-it-out
   c. Scheduled awakenings
   d. Controlled crying
   e. Camping-out
10. The method where parents gradually move farther from the child’s bed each night until they are no longer in the room is:
    a. Controlled crying
    b. Camping-out
    c. Faded bedtime
    d. Cry-it-out
    e. Scheduled awakenings
11. Which of the following is NOT a temporary reaction children may experience when starting sleep training?
    a. Appetite change
    b. Getting sick easily
    c. Crying often
    d. Refusing naps
    e. Feeling sleepy
12. Which statement is TRUE about applying sleep training methods?
    a. The child should sleep in the same room as siblings
    b. Parents should avoid placing toys in the child’s room
    c. Bright room lights do not affect sleep
    d. Having a TV or electronic devices in the bedroom increases night wakings
    e. Room temperature is not important for optimal sleep
13. Recommended total daily sleep (including naps) for ages 4–12 months (AASM):
    a. 6–8 hours
    b. 8–10 hours
    c. 11–12 hours
    d. 12–16 hours
    e. 16–17 hours
14. Which statement is TRUE about bedtime routines?
    a. Eating at night doesn’t require tooth brushing
    b. Bedtime routines support optimal sleep and emotional-behavioral regulation
    c. Children under 5 should not eat before bed even if hungry
    d. Children should not bathe before bed
    e. Bedtime routines can last up to 1 hour
15. Which of the following is NOT a symptom of childhood insomnia?
    a. Difficulty falling asleep
    b. Frequent night awakenings
    c. Snoring
    d. Bedtime procrastination
    e. Difficulty maintaining sleep
16. Which of the following is NOT a positive behavior supporting sleep training?
    a. Sleeping with a favorite doll
    b. Thumb sucking
    c. Humming during sleep
    d. Being rocked or swung to sleep
    e. All of the above
17. Recommended total daily sleep for ages 1–2 years (AASM):
    a. 6–8 hours
    b. 8–10 hours
    c. 11–14 hours
    d. 14–16 hours
    e. 16–17 hours
18. Which statement is FALSE about bedtime routine recommendations?
    a. Regular meal, nap, bedtime, and wake times support optimal sleep
    b. The bedroom can be both a play and rest area
    c. Caffeine (soda, tea, chocolate) should be avoided after lunch
    d. Physical play should be avoided near bedtime
    e. A favorite toy can comfort the child during sleep
19. How long should parents wait before deciding sleep training is ineffective?
    a. 5–7 days
    b. 1–2 weeks
    c. 3–4 weeks
    d. 1–2 months
    e. 3–4 months
20. Which sleep training methods are recommended?
    a. Cry-it-out
    b. Controlled crying
    c. Faded bedtime
    d. Scheduled awakenings
    e. All of the above

**Scoring:**
Correct = 5 points, Incorrect = 0
Good knowledge: ≥70
Poor knowledge: <70

**TOTAL SCORE**

**SLEEP TRAINING BEHAVIOR SURVEY**

Circle the most appropriate answer describing your child’s condition in the past week.

1. **My child …**
   1. Goes to bed at the same time every night (e.g., 8 PM)
   2.
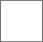
Goes to bed at different times each night
2. **My child …**
   1. Does not eat before bed
   2. Eats before bed

If eats, choose the appropriate options (more than one allowed):

1. Healthy snacks (bread, biscuits, cake)
2. Milk
3. Rice and side dishes
4.
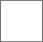
Sweet foods (chocolate, candy, soda)
5. Others:
6. **My child …**
   1. Does not clean before bed
   2. Cleans up before bed

If cleans up, choose one:

1. Bathing
2. Brushing teeth
3.
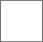
Washing hands and feet
4. Brushing teeth + washing hands and feet
5. Others:
6. **Activities before bed** (choose more than one):
   1. Reading stories
   2. Listening to soft music
   3. Watching TV
   4.
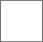
Playing with gadgets
   5. Exercise or other vigorous activity
   6. Others:
7. **My child …**
   1. Needs no specific routine to fall asleep
   2. Needs a specific routine to fall asleep

If yes, choose (more than one allowed):

1. Story reading
2. Singing
3.
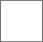
Rocking
4. Breastfeeding
5. Others:
6. **My child …**
   1. Sleeps alone in their room
   2. Shares a room with others (parents, nanny, siblings, etc.)


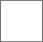
If shares a room:

1. Sleeps alone on a separated bed
2. Shares the bed with parents/siblings
3. **My child’s bedroom …**
   1. Lights are off
   2. Lights are on

**Temperature:**

1. No air cooling
2. Uses air cooling

**If there’s air cooling:**

1. AC
2. Fan
3. Others:

**Toys/entertainment** (choose more than one):

1. None
2. Favorite doll/blanket
3. Television
4. Computer
5.
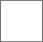
Others:
6. **When my child wakes up crying, I…**
   1. Do not respond
   2. Try to soothe them

If soothe, choose … (more than one allowed)

1. Rocking
2. Singing
3.
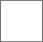
Breastfeeding
4. Others:
5. **I put my child to sleep by ...** (choose more than one)
   1. Feeding milk
   2. Rocking/singing
   3.
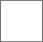
Storytelling
   4. Alternating methods
   5. Doing nothing
6. **In the morning, my child wakes up ...**
   1.
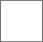
At the same time every day
   2. At different times

**TOTAL SCORE**

**Scoring:**

Good behavior: ≥ 14
Poor behavior: < 14

**Supplementary File 2.** Indonesian Adaptation of The Questionnaire Titled “Knowledge and Behavior of Mothers with Children Aged 3–36 Months Regarding Sleep Training”

**FORMULIR IDENTITAS SUBJEK PENELITIAN**

**Tanggal Pengisian:**

**Tempat Pengisian*:** Puskesmas Kec. Jatinegara / Poliklinik RSIA Bunda / Puskesmas Keboan

1. **Identitas Ibu**

| a. Nama | : | | |
| --- | --- | --- | --- |
| b. Tempat, tanggal lahir | : | | |
| c. Usia | : | | |
| d. Alamat | : | | |
| e. No HP | : | | |
| f. Pendidikan terakhir** : | | 1. SD/sederajat 2. SMP/sederajat 3. SMA/sederajat 4. Diploma/sederajat | 1. S1/sederajat 2. S2/sederajat 3. S3/sederajat 4. Tidak bersekolah |

g. Pekerjaan:

h. Pendapatan keluarga** :**Tempat Tinggal di DKI Jakarta***

- 1. Kurang dari Rp5.000.000/bulan
  2. Rp5.000.000/bulan atau lebih

**Tempat Tinggal di Kabupaten Jombang***

1. Kurang dari Rp 3.000.000/bulan
2. Rp3.000.000/bulan atau lebih
3. **Identitas Anak**
   1. Nama :
   2. Jenis kelamin :
   3. Tempat, tanggal lahir :
   4. Usia :
   5. Anak ke : dari bersaudara

**Keterangan**

- : Coret yang tidak perlu

**: Lingkari pilihan yang sesuai

1. **Lokasi Tidur Anak**

Lingkari dan isi jawaban yang sesuai dengan kondisi tidur anak di rumah! Jawaban dapat dipilih lebih dari 1

1. Ruangan tidur anak saya berada di** :
   1. Kamar tidur anak
   2. Kamar tidur orang tua
   3. Ruang keluarga
   4. Lain-lain, sebutkan
2. Anak saya tidur di dalam **satu ruangan** bersama**:
   1. Sendiri
   2. Ibu
   3. Ayah
   4. Saudara kandung, berjumlah orang
   5. Lain-lain, sebutkan , berjumlah orang

**Total jumlah orang dalam satu ruangan** = orang

1. Anak saya tidur di atas **satu tempat tidur** bersama**:
   1. Sendiri
   2. Ibu
   3. Ayah
   4. Saudara kandung, berjumlah orang
   5. Lain-lain, sebutkan , berjumlah orang

**Total jumlah orang dalam satu tempat tidur** = orang

**Keterangan**

- : Coret yang tidak perlu

**: Lingkari pilihan yang sesuai

**PERTANYAAN PENGETAHUAN**

Pilihlah 1 (satu) jawaban yang benar dari beberapa pertanyaan berikut!

1. Latihan tidur merupakan strategi yang dapat diterapkan untuk mendukung tidur mandiri pada anak. Di bawah ini merupakan pernyataan yang **BENAR** …
   1. Latihan tidur dapat mengatasi masalah tidur pada anak
   2. Latihan tidur pada anak dapat membantu menerapkan rutinitas tidur yang konsisten
   3. Penerapan latihan tidur tidak seragam untuk semua anak karena menyesuaikan dengan kecocokan masing-masing anak dan preferensi orang tua
   4. Rasa khawatir dari orang tua ketika mendengar anak menangis dapat menghambat keberhasilan latihan tidur
   5. Semua benar
2. Kapan usia terbaik bagi anak untuk mulai dilakukan latihan tidur?
   1. 1-3 bulan
   2. 4-6 bulan
   3. 7-9 bulan
   4. 10-12 bulan
   5. 1-3 tahun
3. Di bawah ini **BUKAN** merupakan tanda kesiapan anak dalam memulai latihan tidur …
   1. Anak tidak mudah menangis jika jauh dari orang tua
   2. Anak mulai memiliki siklus tidur/bangun secara teratur
   3. Anak mampu menenangkan diri sendiri untuk tidur
   4. Anak dapat tidur sepanjang malam tanpa perlu bangun untuk menyusu
   5. Anak telah memiliki sistem pengaturan tidur di dalam tubuh yang matang
4. Tujuan latihan tidur adalah untuk membantu anak dalam memiliki tidur mandiri melalui perilaku positif. Manakah di bawah ini yang termasuk perilaku positif yang menunjang latihan tidur pada anak?
   1. Menimang anak sebelum tidur
   2. Menyanyikan lagu sebelum tidur
   3. Menyusui anak sebelum tidur
   4. Anak berbaring di samping orang tua
   5. Anak tidur dengan boneka kesayangan
5. Manakah pernyataan yang **BENAR** mengenai tidur pada anak ?
   1. Anak yang dibiarkan menangis ketika tidur dapat mengalami gangguan emosional di kemudian hari
   2. Tidak ada bukti yang menunjukkan bahwa latihan tidur dapat memperbaiki kesehatan fisik dan mental orang tua
   3. Anak sebaiknya disusui hingga tertidur agar anak dapat tidur lebih nyenyak
   4. Anak harus ditimang oleh orang tua hingga tertidur agar anak tidak mudah terbangun di malam hari
   5. Tidak ada jawaban benar
6. Metode latihan tidur yang dilakukan dengan menidurkan bayi, lalu meninggalkan kamar tanpa memeriksa bayi sampai waktu yang ditentukan pada keesokan paginya dikenal sebagai metode …
   1. Menunda waktu tidur anak *(faded bedtime)*
   2. Membiarkan anak menangis *(cry-it-out)*
   3. Membangunkan anak secara terjadwal *(scheduled awakenings)*
   4. Mengendalikan waktu menangis anak *(controlled crying)*
   5. Memberi jarak dengan area tidur anak *(camping-out*)
7. Metode latihan tidur yang dilakukan dengan orang tua menidurkan bayi, lalu orang tua meninggalkan kamar, diikuti dengan memeriksa bayi setiap 3 menit, 5 menit, 10 menit, dengan interval yang lebih panjang dalam waktu satu minggu merupakan contoh penerapan dari metode …
   1. Membiarkan anak menangis *(cry-it-out)*
   2. Menunda waktu tidur anak *(faded bedtime)*
   3. Membangunkan anak secara terjadwal *(scheduled awakenings)*
   4. Mengendalikan waktu menangis anak *(controlled crying)*
   5. Memberi jarak dengan area tidur anak *(camping-out)*
8. Metode latihan tidur yang dilakukan dengan menunda jadwal tidur pada anak dan menerapkan rutinitas tidur pada anak dengan harapan anak dapat tidur lebih cepat dikenal dengan metode …
   1. Memberi jarak dengan area tidur anak *(camping-out)*
   2. Membangunkan anak secara terjadwal *(scheduled awakenings)*
   3. Menunda waktu tidur anak (faded bedtime)
   4. Mengendalikan waktu menangis anak *(controlled crying)*
   5. Membiarkan anak menangis *(cry-it-out)*
9. Metode latihan tidur yang dilakukan dengan membangunkan anak (seperti dengan cara menggelitik kakinya) sekitar 15-30 menit sebelum waktu biasanya anak terbangun, dengan harapan anak dapat sedikit terbangun dan kembali tidur dalam hitungan detik merupakan contoh dari penerapan metode …
   1. Menunda waktu tidur anak *(faded bedtime)*
   2. Membiarkan anak menangis (*cry-it-out)*
   3. Membangunkan anak secara terjadwal *(scheduled awakenings)*
   4. Mengendalikan waktu menangis anak *(controlled crying)*
   5. Memberi jarak dengan area tidur anak *(camping-out)*
10. Metode latihan tidur yang dilakukan dengan orang tua tetap berada di kamar anak ketika anak tertidur, kemudian berjarak semakin jauh setiap malamnya hingga orang tua tidak lagi berada di kamar anak ketika anak tertidur, dikenal dengan metode …
    1. Mengendalikan waktu menangis anak *(controlled crying)*
    2. Memberi jarak dengan area tidur anak *(camping-out)*
    3. Menunda waktu tidur anak *(faded bedtime)*
    4. Membiarkan anak menangis *(cry-it-out)*
    5. Membangunkan anak secara terjadwal *(scheduled awakenings)*
11. Di bawah ini **BUKAN** merupakan respon sementara yang dapat dialami anak ketika metode latihan tidur mulai diterapkan…
    1. Anak mengalami perubahan nafsu makan
    2. Anak mudah sakit
    3. Anak mudah menangis
    4. Anak tidak mau tidur siang
    5. Anak mudah mengantuk
12. Manakah pernyataan yang **BENAR** mengenai penerapan metode latihan tidur pada anak? …
    1. Anak sebaiknya tidur di dalam satu kamar dengan saudara kandung
    2. Orang tua sebaiknya menghindari meletakkan boneka atau mainan kesayangan di dalam kamar anak
    3. Cahaya lampu terang di dalam kamar tidak akan mempengaruhi kondisi tidur anak
    4. Meletakkan TV atau perangkat elektronik lainnya di dalam kamar dapat meningkatkan kejadian terbangun di malam hari pada anak
    5. Suhu kamar tidak memiliki peran penting dalam mendukung tidur yang optimal pada anak
13. Durasi tidur anak yang cukup dalam 24 jam (termasuk tidur siang) secara teratur bagi anak berusia 4-12 bulan *(American Academy of Sleep Medicine*)
    1. 6-8 jam
    2. 8-10 jam
    3. 11-12 jam
    4. 12-16 jam
    5. 16-17 jam
14. Pernyataan di bawah ini yang **BENAR** berkaitan dengan implementasi rutinitas sebelum tidur pada anak …
    1. Makan sedikit di malam hari tidak harus diikuti dengan rutinitas menyikat gigi sebelum tidur
    2. Penerapan rutinitas sebelum tidur bagi anak tidak hanya mendukung tidur optimal, tetapi juga dapat membantu dalam regulasi emosional-perilaku
    3. Anak berusia di bawah 5 tahun tidak dianjurkan untuk makan sebelum tidur meskipun merasa lapar
    4. Anak tidak dianjurkan untuk mandi sebelum tidur
    5. Rutinitas sebelum tidur dapat dilakukan selama hingga 1 jam
15. Berikut ini yang **BUKAN** merupakan gejala gangguan perilaku tidur insomnia pada anak…
    1. Anak mengalami kesulitan untuk mulai tertidur
    2. Anak sering terbangun di malam hari
    3. Anak terdengar mengorok ketika tidur
    4. Anak menunda waktu tidur malam
    5. Anak sulit mempertahankan tidur
16. Tujuan latihan tidur adalah untuk membantu anak dalam memiliki tidur mandiri melalui perilaku positif. Di bawah ini yang **BUKAN** termasuk perilaku positif untuk menunjang latihan tidur pada anak …
    1. Anak tidur bersama boneka kesayangan
    2. Anak mengisap jempol ketika tidur
    3. Anak mengeluarkan suara seperti mendengung ketika tidur
    4. Anak ditimang dan diayun hingga tertidur
    5. Semua jawaban benar
17. Durasi tidur anak yang cukup dalam 24 jam (termasuk tidur siang) secara teratur bagi anak berusia 1-2 tahun *(American Academy of Sleep Medicine)* …
    1. 6-8 jam
    2. 8-10 jam
    3. 11-14 jam
    4. 14-16 jam
    5. 16-17 jam
18. Pernyataan di bawah ini yang **SALAH** mengenai anjuran rutinitas sebelum tidur pada anak…
    1. Jadwal makan, tidur siang, tidur malam, dan bangun pagi yang teratur pada anak berkaitan dengan tidur yang optimal
    2. Kamar tidur dapat dijadikan tempat istirahat sekaligus bermain bagi anak
    3. Konsumsi kafein (soda, teh, cokelat) sebaiknya dihindari setelah waktu makan siang
    4. Anak sebaiknya menghindari olahraga dan permainan aktif lainnya mendekati jam tidur
    5. Boneka kesayangan dapat memberikan kenyamanan bagi anak untuk tidur
19. Berapa lama orang tua dapat memastikan bahwa penerapan metode latihan tidur pada anak belum memberikan perubahan atau hasil yang diinginkan?
    1. 5-7 hari
    2. 1-2 minggu
    3. 3-4 minggu
    4. 1-2 bulan
    5. 3-4 bulan
20. Berikut ini merupakan metode latihan tidur yang direkomendasikan…
    1. Membiarkan anak menangis *(cry-it-out)*
    2. Mengendalikan waktu menangis anak *(controlled crying)*
    3. Menunda waktu tidur anak *(faded bedtime)*
    4. Menjadwalkan waktu terbangun anak *(scheduled awakenings)*
    5. Semua jawaban benar

**NILAI TOTAL**

**PENILAIAN**

1 soal terjawab benar = 5 poin; 1 soal terjawab salah = 0 poin **Interpretasi:** Pengetahuan baik jika nilai total ≥70 Pengetahuan kurang jika nilai total <70

**SURVEY PERILAKU LATIHAN TIDUR**

Lingkari pilihan yang paling sesuai dengan kondisi dalam 1 (satu) minggu terakhir!

1. **Anak saya …**
   1. Tidur pada jam yang sama setiap malam (misalkan jam 20.00)
   2.
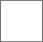
Tidur di jam yang berbeda-beda setiap malam
2. **Anak saya …**
   1. Tidak makan sebelum tidur
   2. Makan sebelum tidur

Jika makan, lingkari pilihan yang paling sesuai (**pilihan boleh lebih dari 1**)

1. Makan camilan sehat (misalkan roti, biskuit, bolu)
2. Minum susu
3. Makan nasi dan lauk pauk
4.
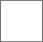
Makan makanan manis (misalkan cokelat, permen, soda)
5. Lainnya, sebutkan
6. **Anak saya …**
   1. Tidak membersihkan badan sebelum tidur
   2. Membersihkan badan sebelum tidur

Jika membersihkan badan, **pilih 1 jawaban** yang paling sesuai

1. Mandi
2. Menggosok gigi
3.
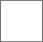
Mencuci tangan dan kaki
4. Menggosok gigi, mencuci tangan dan kaki
5. Lainnya, sebutkan
6. **Aktivitas anak saya sebelum tidur …** (pilihan boleh lebih dari 1)
   1. Membaca dongeng
   2. Mendengarkan musik lembut
   3. Menonton TV
   4.
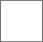
Bermain *gadget*
   5. Olahraga dan aktivitas berat lainnya
   6. Lainnya, sebutkan
7. **Anak saya …**
   1. Tidak perlu melakukan rutinitas apapun agar bisa tertidur
   2. Perlu melakukan rutinitas tertentu agar bisa tertidur

Jika ada rutinitas tertentu, lingkari pilihan yang sesuai **(pilihan boleh lebih dari 1)**

1. Dibacakan buku / dongeng hingga tertidur
2. Dinyanyikan lagu hingga tertidur
3.
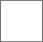
Ditimang hingga tertidur
4. Disusui hingga tertidur
5. Lainnya, sebutkan
6. **Anak saya …**
   1. Tidur sendiri di kamarnya
   2. Tidur di kamar bersama orang lain (orang tua, pengasuh, saudara, dll)


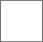
 Jika anak tidur di kamar bersama orang lain, anak saya tidur ……

1. Sendiri di kasurnya
2. Bersama orang tua atau saudara
3. **Ruangan tidur anak saya …**
   1. Lampu dimatikan
   2. Lampu menyala

**Ruangan tidur anak saya …**

1. Tidak menggunakan pendingin ruangan
2. Menggunakan pendingin ruangan

Jika menggunakan pendingin ruangan, sebutkan pendingin ruangan ...

1. *Air condition* (AC)
2. Kipas angin
3. Lainnya, sebutkan

**Mainan/hiburan di ruangan tidur anak saya … (pilihan boleh lebih dari 1)**

1. Tidak ada mainan / hiburan di ruangan tidur anak saya
2. Boneka / selimut kesayangan anak
3.
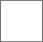
Televisi
4. Komputer
5. Lainnya, sebutkan
6. **Saat anak saya menangis karena terbangun dari tidur, saya…**
   1. Tidak merespon
   2. Mencoba menenangkan anak

Jika mencoba menenangkan, saya melakukan … **(pilihan boleh lebih dari 1)**

1. Menimang
2. Menyanyikan lagu
3.
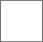
Menyusui
4. Lainnya, sebutkan
5. **Saya menidurkan anak saya dengan cara ... (pilihan boleh lebih dari 1)**
   1. Menyusui / memberikan susu
   2. Menimang / menyanyikan lagu
   3.
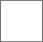
Mendongeng
   4. Berganti-gantian cara menidurkan anak
   5. Tidak melakukan apa-apa
6. **Anak saya pagi hari bangun ...**
   1.
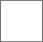
Pada jam yang sama setiap harinya
   2. Pada jam yang berbeda-beda

**NILAI TOTAL**

**Interpretasi:**

Perilaku baik jika nilai total ≥ 14 Perilaku kurang jika nilai total < 14

**Supplementary File 3.** Validity and Reliability Analyses of Knowledge Domain (N = 35)

| **Item** | **Mean** | **SD** | **Corrected Item-Total Correlation** | **Cronbach’s α if Item Deleted** |
| --- | --- | --- | --- | --- |
| P1 | 2.43 | 2.54 | .073 | .744 |
| P2 | 1.43 | 2.29 | –.114 | .756 |
| P3 | 1.86 | 2.45 | .105 | .741 |
| P4 | 1.57 | 2.36 | .362 | .720 |
| P5 | 2.43 | 2.54 | .325 | .723 |
| P6 | 1.71 | 2.41 | .195 | .733 |
| P7 | 3.14 | 2.45 | .338 | .722 |
| P8 | 3.00 | 2.49 | .590 | .699 |
| P9 | 3.57 | 2.29 | .164 | .735 |
| P10 | 3.86 | 2.13 | .458 | .713 |
| P11 | 2.57 | 2.54 | .559 | .702 |
| P12 | 1.71 | 2.41 | .296 | .725 |
| P13 | 2.57 | 2.54 | .647 | .693 |
| P14 | 3.71 | 2.22 | .248 | .729 |
| P15 | 3.14 | 2.45 | .271 | .727 |
| P16 | 1.29 | 2.22 | .202 | .732 |
| P17 | 2.71 | 2.53 | .492 | .708 |
| P18 | 1.29 | 2.22 | .422 | .716 |
| P19 | 3.14 | 2.45 | –.016 | .750 |
| P20 | 3.29 | 2.41 | .400 | .717 |
| **Scale Mean = 50.43** | **Scale SD = 19.53** |  | **Cronbach’s α = .735** |  |

**Supplementary File 4.** Validity and Reliability Analyses of Behavior Domain (N = 70)

| **Item** | **Mean** | **SD** | **Corrected Item-Total Correlation** | **Cronbach’s α if Item Deleted** |
| --- | --- | --- | --- | --- |
| P1 | 1.47 | 2.06 | .421 | .794 |
| P2 | 1.11 | 1.70 | –.043 | .828 |
| P3 | 1.61 | 1.81 | .125 | .817 |
| P4 | 1.09 | 1.78 | .435 | .792 |
| P5 | 1.40 | 2.09 | .479 | .788 |
| P6 | 0.94 | 1.88 | .455 | .791 |
| P7A | 1.99 | 2.10 | .608 | .775 |
| P7B | 1.99 | 2.03 | .683 | .768 |
| P7C | 2.23 | 2.11 | .595 | .776 |
| P8 | 1.94 | 2.44 | .767 | .754 |
| P9 | 2.06 | 1.96 | .470 | .789 |
| P10 | 1.20 | 1.80 | .346 | .800 |
| **Scale Mean = 19.03** | **Scale SD = 13.46** |  | **Cronbach’s α = .805** |  |
